# Addressing the fistula treatment gap and rising to the 2030 challenge

**DOI:** 10.1002/ijgo.13033

**Published:** 2020-01-13

**Authors:** Gillian Slinger, Lilli Trautvetter

**Affiliations:** ^1^ Fistula Surgery Training Initiative FIGO (International Federation of Gynecology and Obstetrics) London UK

**Keywords:** Capacity building, FIGO, Fistula, Holistic care, Obstetric fistula, Surgery, Sustainable Development Goals (SDGs), Training

## Abstract

Obstetric fistula is a neglected public health and human rights issue. It occurs almost exclusively in low‐resource regions, resulting in permanent urinary and/or fecal incontinence. Although the exact prevalence remains unknown, it starkly outweighs the limited pool of skilled fistula surgeons needed to repair this childbirth injury. Several global movements have, however, enabled the international community to make major strides in recent decades. FIGO's Fistula Surgery Training Initiative, launched in 2012, has made significant gains in building the capacity of local fistula surgeons to steadily close the fistula treatment gap. Training and education are delivered via FIGO and partners’ Global Competency‐based Fistula Surgery Training Manual and tailored toward the needs and skill level of each trainee surgeon (FIGO Fellow). There are currently 62 Fellows from 22 fistula‐affected countries on the training program, who have collectively performed over 10 000 surgical repairs. The initiative also contributes to the UN's Sustainable Development Goals (1, 3, 5, 8, 10, and 17). The UN's ambitious target to end fistula by 2030 will be unobtainable unless sufficient resources are mobilized and affected countries are empowered to develop their own sustainable eradication plans, including access to safe delivery and emergency obstetric services.

## Abstract 2

FIGO's methodological training approach is making vital contributions to closing the global fistula treatment gap and calls for more action and resources to end fistula by 2030.

## 1 INTRODUCTION

Obstetric fistula, caused by unrelieved obstructed labor, results in a hole between the vagina and the bladder and/or rectum. The condition leads to chronic incontinence and continues to be a serious public health concern in sub‐Saharan Africa and Asia. Fistulae occur in contexts with a high maternal mortality ratio^1^, ^2^ where expectant mothers do not have access to emergency obstetric care and other essential maternal/reproductive health services. The condition causes severe psychosocial, physical, and economic distress and has been classed as a violation of internationally recognized human rights.^3^, ^4^, ^5^, ^6^

It is estimated that around two million women and girls in 60 under‐resourced countries currently live with a fistula and that between 50 000 and 100 000 new cases develop each year.^7^ The main curative treatment remains corrective surgery; however, due to a global shortage of fistula surgeons, current treatment rates indicate that only one woman in 50 receives a fistula repair.^8^

Key global movements to help eradicate obstetric fistula include UNFPA's Campaign to End Fistula (2003); the International Society of Obstetric Fistula Surgeons (ISOFS, 2008); the Global Fistula Map^8^ established by Direct Relief, Fistula Foundation, and UNFPA; and the UN's International Day to End Obstetric Fistula (2013), which is observed on May 23 each year. While these efforts have been invaluable in putting obstetric fistula on the global health agenda, a systematic and standardized training approach was still required to train the next generation of competent fistula surgeons and to meet the treatment gap. FIGO (the International Federation of Gynecology and Obstetrics), together with partners identified this need and decided to act.

## 2 BACKGROUND

The two long‐standing factors impeding fistula treatment work were the absence of a widely agreed curriculum for fistula surgeons, and a coordinated international training program for fistula surgeons. To overcome these two obstacles, FIGO convened leading authorities from the fistula world (including expert surgeons and international agencies) to address the global treatment gap with a two‐tiered approach: education and training.

- In 2011, after numerous stakeholder meetings, releasing the world's first standardized curriculum—FIGO and partners’ Global Competency‐based Fistula Surgery Training Manual—to train fistula surgeons.^9^
- In 2012, launching the Fistula Surgery Training Initiative^10^—an ambitious multiyear program with the aim of training more fistula surgeons (using the Competency‐based Manual) to considerably increase the number of skilled fistula surgeons in affected countries so that significantly more women receive treatment.

After introducing the new Training Manual in workshops to more than 50 fistula surgeons and trainers in Africa and Asia, five busy, well‐functioning fistula treatment facilities were identified as FIGO Training Centers in Ethiopia, Tanzania, Kenya, and Nigeria (two centers). Then, following strict selection criteria (Box 1), in collaboration with ministries of health, hospital management teams, and multiple partners, FIGO started to recruit local fistula surgeon trainees (Fellows) on to the program, focusing exclusively on building national capacity to boost fistula treatment work in high‐burden countries.

Using the Training Manual, a Fellow's skills in fistula surgery (and care of affected women) are methodically developed over time and through three ascending levels of competency: standard, advanced, and expert. This takes place with a series of training placements in the identified training centers, as well as coaching visits from a FIGO Trainer in Fellows’ home environments. This process complements Fellows’ existing clinical roles, while aiming to cause minimal disruption to their existing work and hospital schedules.

As Fellows come from various medical disciplines, including obstetrics and gynecology, urology, and general surgery, they have widely differing professional responsibilities, of which fistula treatment is just one component. A flexible training approach is therefore essential and can be tailored to meet the needs of each trainee, allowing additional study or placements away from usual work settings without the Fellow losing their place on the training program. This supple training system accommodates Fellows’ career progression while ensuring their continued development as a fistula surgeon, thereby optimizing investment in the initiative and—directly in line with the aim of the program—ensuring fistula treatment is provided for substantially more women in the long term.

Box 1 **Selection criteria for FIGO Fellows.**

1 • Must be a qualified physician with a minimum of 3 years’ surgical experience

• Must originate from and be in full‐time clinical work in a fistula‐affected country

• Must have a proven track record and strong commitment to caring for women with fistula

• Must be available to undergo 6 weeks of initial training then subsequent coaching

• Assurance must be provided by the Ministry of Health and hospital management that the Fellow will be able to continue providing fistula treatment after the initial training placement on return to their home country

• Must be committed to the care of women who have incurred obstetric fistula and to upholding women's basic rights to privacy, dignity, safety, and self‐determination

• Must be prepared to apply, immediately and on a long‐term basis, the skills gained during training placements/coaching sessions and upon returning to their home environment

• Robust references must be provided directly to FIGO by recognized fistula surgeon(s)

Note: The selection decision is taken jointly by the FIGO team with the appointed training center

As the project has grown, two objectives have been developed that correspond to both short‐ and long‐term goals:

1. Short‐term objective

To strengthen fistula treatment capacity in each affected country by developing an appropriate pool of trained, competent fistula surgeons who can accelerate efforts to address the fistula treatment gap and thereby treat significantly more women in that country.

2 Long‐term objective

To develop a group of Fellows to Trainer level—to reinforce the pool of FIGO trainers—to scale up global treatment efforts by helping to train more fistula surgeons (and holistic care teams) in their own country and internationally.

## 3 ACHIEVEMENTS

**Figure 1:**
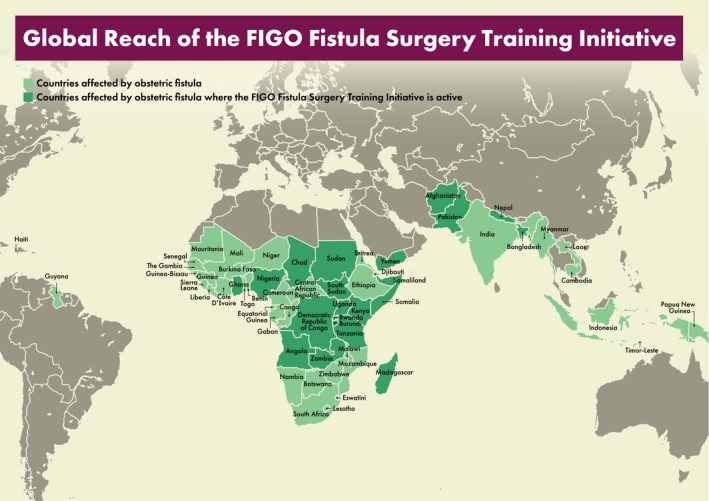
Global reach of the FIGO fistula surgery training initiative.

Great progress has been made since the initiative began, with some notable achievements listed below:

- 62 Fellows recruited from 22 high‐burden countries (Table 1 and Fig. 1).
- Collectively the Fellows have performed more than 10 000 fistula surgeries to date, with a success rate of 84%.
- Tailored coaching visits provided to over 30 Fellows, helping them to refine their skills.
- Certification mechanism set up, in line with the Training Manual, through which more than 50 Fellows have attained the standard level of competency in fistula surgery (of whom many are now working toward the advanced level); 13 Fellows have attained the advanced level of competency in fistula surgery (of whom several are now working toward the expert level).
- 9 multidisciplinary healthcare teams admitted for training in holistic care of fistula patients.
- 50 high‐quality fistula instrument sets and more than 30 surgical head torches supplied to Fellows via the pioneering FIGO–Medical Aid International Equipment Alliance.^11^
- Comprehensive Kaizen e‐Portfolio monitoring and evaluation system^12^ established to track Fellows’ progress and program impact. This allows Fellows and trainers to submit information directly, including training assessment forms, and quarterly data containing Fellows’ repair numbers and surgical outcomes. This method also creates robust feedback loops, helping the project team to make data‐driven decisions and to consistently weave in lessons learned.
- Expert Advisory Group (EAG) created, comprised of the project team and FIGO trainers, to provide consensus and expert guidance to the evolving program, which has grown significantly in recent years both in terms of size and complexity.
- Substantial increase in requests being received from sponsors, including national and international nongovernmental organizations and UN entities, to train more Fellows and care teams.
- Vast network of collaborating partners developed and being coordinated, including fistula treatment facilities, training centers, authorities, and patient recruitment teams in Africa and Asia.

**Table 1:** Country of origin of FIGO Fellows currently on the training program (October 2019)

Country	Number of Fellows
1. Afghanistan	2
2. Angola	1
3. Bangladesh	3
4. Burundi	1
5. Chad	2
6. Democratic Republic of Congo	4
7. Gambia	2
8. Ghana	4
9. Kenya	6
10. Madagascar	1
11. Nepal	4
12. Nigeria	13
13. Pakistan	1
14. Rwanda	3
15. Somalia	2
16. Somaliland	1
17. Sudan	1
18. South Sudan	1
19. Tanzania	2
20. Uganda	2
21. Yemen	2
22. Zambia	4
Total	62

## 4 MEETING THE SUSTAINABLE DEVELOPMENT GOALS AND THE 10‐YEAR COUNTDOWN

Since the launch of the UN's Sustainable Development Goals (SDGs) in 2015,^13^ FIGO's Fistula Surgery Training Initiative has been making crucial contributions to the Global Development Agenda 2030. While the program supports the advancement of women's sexual and reproductive health, rights, and prosperity in some of the most under‐served and marginalized communities, contributions are particularly relevant to SDG 3 (good health and well‐being pertaining to women's sexual and reproductive health and rights, as well as newborn, child, and adolescent health) and SDG 5 (gender equality). Furthermore, as the Initiative is providing life‐changing care and enhanced life opportunities for some of the poorest, most disadvantaged women on the planet, it also plays a role in achieving SDG 1 (no poverty), SDG 8 (decent work and economic growth), SDG 10 (reduced inequalities), and SDG 17 (partnerships).

At the end of 2018, the UN challenged its member states to end obstetric fistula by 2030.^14^ Without question, even though the global eradication of obstetric fistula is long overdue, achieving this goal within a decade is extremely ambitious, particularly with the current infrastructure, and limited human as well as financial resources.

It has long been known that the key to eradicating obstetric fistula is to change the multiple systems currently failing so many women. To do this, each fistula‐affected country should develop its own eradication strategy that is costed, well‐resourced, time‐bound, and built into a framework supporting gender equality and the socioeconomic development of women and girls.

Furthermore, it is necessary that the implementation of such strategies takes place at a national, regional, and local level, for which governmental support and leadership are mandatory to ensure sustained success. Any such strategies are to be carefully monitored and will ideally encompass awareness raising, prevention of (first and subsequent) obstetric fistula, availability of treatment services, and collaborative partnerships.

Integrated into a human rights‐based approach, awareness raising and prevention of obstetric fistula would preferably start at a preadolescent age so that girls, women, and men are empowered to make informed decisions. Moreover, affordable, accessible, and safe emergency obstetric services are required so that expectant mothers do have a choice and can prepare accordingly.^13^, ^14^

Fistula treatment networks and referral systems also need to expand so that even patients in the most remote locations have the chance of a surgical repair by a trained, competent fistula surgeon. In addition, countries with decades of experience and dedicated specialist fistula task forces should be encouraged to share their rich expertise with those that are further behind, to avoid delaying progress.

## 5 LESSONS LEARNED AND FUTURE PRIORITIES

**Figure 2:**
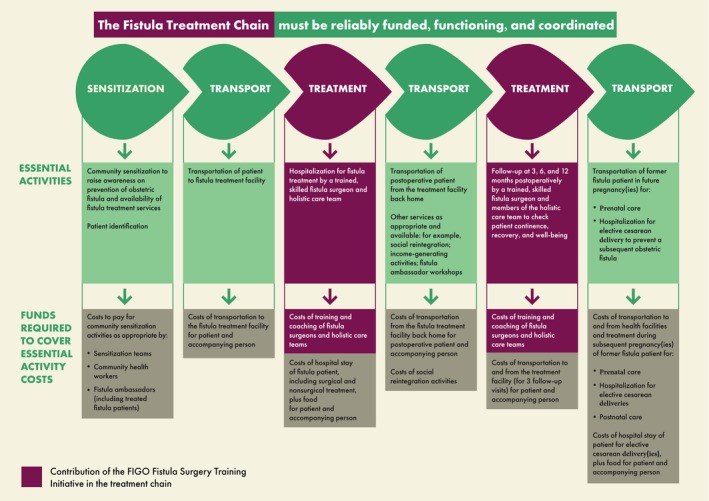
The fistula treatment chain.

Training more fistula surgeons—and multidisciplinary health teams—is crucial to helping women with fistulae, but it must be part of a system‐wide approach, “the fistula treatment chain” (G. Slinger, oral communication, December 2018), with the involvement of local authorities and multiple partners to ensure quality care is available and accessible for affected women. Furthermore, the vital training component (of fistula surgeons and holistic care teams) is an essential link in the treatment chain. But to make certain that more affected women receive treatment in a sustainable manner, all links in the chain must be reliably funded, functioning, and coordinated. Experience repeatedly shows, through collaborative efforts, that such mechanisms make the best use of limited resources and bring in high patient numbers, yet it only needs one link in the fragile chain to fail (e.g. through lack of funding) and the whole system breaks down (Fig. 2).

Developing competencies in fistula surgery requires appropriate time and funding. This is not a single training event, but an ongoing process to build skills gradually, while allowing for changes in Fellows’ circumstances and making every effort to integrate fistula treatment activities into local health programs. While striving to close the fistula treatment gap, Fellows are encouraged to do more fistula repairs with supportive measures. But they are never pressurized to increase their repair numbers “at any price,” as this could potentially compromise patient safety, disrupt local health schedules, and lead to data distortions.

Evidence shows that combined efforts of diverse surgical disciplines and broader training methods can certainly enhance fistula repair work.^15^ However, because of the complexity of fistula surgery, and the long‐standing neglect of women with the condition—making them one of the hardest to reach, most vulnerable groups—it is essential to maintain a focused fistula community and a focused training program, such as the FIGO Fistula Surgery Training Initiative, to prevent lapses in progress and to close the global treatment gap.

In direct collaboration with partners at all levels, and with ongoing support from the EAG, in the next phase, the training program aims to:

- Admit new Fellows/health teams from neglected countries.
- Provide ongoing training/coaching for existing Fellows to help them attain advanced and expert levels, then to become FIGO trainers with the ability to train many others and to adopt the FIGO and partners’ training curriculum (as appropriate) in their country and region.
- Identify additional training centers in francophone Africa and Asia.
- Convene surgical workshops for Fellows and trainers.
- Continue refining and supplying specialized fistula equipment to Fellows, as well as making the materials available at reduced rates to the broader fistula community through the FIGO–Medical Aid International Equipment Alliance.
- Strengthen the fistula treatment chain in collaboration with partners.
- Develop more fistula educational materials including best practice guidelines and an updated edition of the Fistula Surgery Training Manual (including English, French, and Portuguese versions).
- Identify research gaps to reinforce the evidence base for all issues relating to fistula.

## 6 CONCLUSION

Expanding the limited pool of fistula surgeons to provide life‐transforming fistula repairs to women in some of the world's most neglected contexts, FIGO's Fistula Surgery Training Initiative is clearly demonstrating the effectiveness of its two‐tiered approach through education and training. With the indispensable support of the dedicated FIGO Fellows, trainers, and EAG, plus an extensive network of collaborating partners, the program is rising to the challenge and making a significant contribution to bridging the fistula treatment gap, and to the Global Development Agenda 2030.

Although progress has undeniably been made in the last 20 years to bring attention to obstetric fistula, the fistula community has a daunting decade ahead if the SDG target to end fistula by 2030 is to be met. With the current set‐up, and woefully insufficient funding across the sector, this is a monumental challenge and bold steps are required to move nearer this goal. FIGO calls to action governments to take decisive measures by developing fistula elimination plans, mobilizing resources, and addressing the underlying drivers perpetuating social, economic, and gender disparities leading to obstetric fistula. Although the 2030 target appears far from achievable, it nevertheless represents a unique opportunity that should be embraced and not ignored, so that those women who are currently furthest behind are not failed.

More information about the program and the Fistula Surgery Training Initiative Newsletter are available at: https://www.figo.org/fistula.

## AUTHOR CONTRIBUTIONS

GS and LT contributed equally to the conception and design of the article. Both authors drafted the work, revised it critically for intellectual content, and provided final approval of the submitted manuscript.

## CONFLICTS OF INTEREST

The authors have no conflicts of interest.
